# Puerarin ameliorates non-alcoholic fatty liver disease by inhibiting lipid metabolism through FMO5

**DOI:** 10.3389/fphar.2024.1423634

**Published:** 2024-07-11

**Authors:** Zhaoyi Li, Wenjing Cao, Yuxuan Zhang, Shanglei Lai, Yingyan Ye, Jianfeng Bao, Ai Fu

**Affiliations:** ^1^ Institute of Hepatology and Epidemiology, Affiliated Hangzhou Xixi Hospital, Zhejiang Chinese Medical University, Hangzhou, Zhejiang, China; ^2^ Department of Hepatology, Affiliated Hangzhou Xixi Hospital, Zhejiang Chinese Medical University, Hangzhou, Zhejiang, China; ^3^ School of Public Health, Zhejiang Chinese Medical University, Hangzhou, Zhejiang, China; ^4^ Department of Medical Research Center, Shaoxing People’s Hospital, Shaoxing, Zhejiang, China; ^5^ Hangzhou Medical College Affiliated Lin’an People’s Hospital, The First People’s Hospital of Hangzhou Lin’an District, Hangzhou, China

**Keywords:** puerarin, non-alcoholic fatty liver disease, FMO5, lipid metabolism, oxidative stress

## Abstract

**Introduction:** *Pueraria lobata* is traditionally used in China for treatment of non-alcoholic fatty liver disease (NAFLD). Puerarin, a functional drug extracted from *Pueraria lobata*, features a pharmacological activity. The present study aims to investigate the effect of puerarin intervention on NAFLD.

**Methods:** We established an NAFLD mouse model using a high-fat diet with 60% fat and evaluated the impact of puerarin intervention.

**Results and discussion:** Our results demonstrate that puerarin intervention significantly ameliorates lipid accumulation and protects the liver from high-fat-induced damage while reducing oxidative stress levels in the liver. Furthermore, puerarin intervention significantly downregulates the transcription levels of acetyl-CoA carboxylase (ACC1) in the liver. It also upregulates the transcription levels of carnitine palmitoyltransferase 1 (CPT1), peroxisome proliferator-activated receptor alpha (PPARα), and peroxisome proliferators-activated receptor γ coactivator alpha (PGC1α), which are related to oxidation. Furthermore, we demonstrated that flavin-containing monooxygenase (FMO5) was involved in the protective effect of puerarin against NFALD. In conclusion, the present study demonstrated the beneficial effect of puerarin on NAFLD and showed that puerarin could prevent liver injury and lipid accumulation caused by NAFLD via activating FMO5. These findings provide a new theoretical basis for applying puerarin as a therapeutic agent for NAFLD.

## 1 Introduction

Consumption of a high-fat diet is a major public health problem that can lead to various diseases of the digestive system and damage to several organs, including the liver and gastrointestinal tract (Tong et al., 2021). The liver plays an essential role in lipid metabolism, and prolonged high-fat diet consumption can lead to NAFLD, characterized by steatosis with or without mild inflammation (Powell et al., 2021). NAFLD is now recognized as a contributing factor to liver cirrhosis and liver cancer (Paternostro and Trauner, 2022). The pathophysiology of NAFLD is complex, with various disease phenotypes and a series of cascading events that pose significant challenges for treatment (Stefan et al., 2019). This has led researchers to search for new and effective targeted therapies to prevent and treat NAFLD.

The imbalance of energy metabolism in the liver is the essence of NAFLD. It impairs the synthesis, transport, and oxidation of various energy metabolism substances such as glucose and fatty acids (Loomba et al., 2021). Consequently, excess triglycerides (TGs) that form from nutrients cannot be transported into white fat for storage, accumulating excessive TG in the liver and resulting in NAFLD (Ipsen et al., 2018). Several metabolic enzymes are involved in this process. FMO5 is a metabolic enzyme closely associated with fatty acid metabolism and is highly expressed in the liver (Veeravalli et al., 2022).

Lingjian Kong et al. reported that FMO5 levels were significantly reduced in alcoholic fatty liver disease (Kong et al., 2021). The study also found that inhibition of FMO5 led to increased levels of phospho-nuclear factor-κB (p-NF-κB), oxidative stress, and production of inflammatory factors, ultimately promoting alcohol-induced liver injury. Additionally, research has demonstrated that FMO5 regulates glucose homeostasis and epididymal fat deposition (Scott et al., 2017; Veeravalli et al., 2022). Therefore, FMO5 may act as a regulator of metabolic stability and may be a potential target for treating NAFLD.

Puerarin is a compound extracted from the traditional Chinese medicine *Pueraria lobata*. It belongs to the isoflavone family and has antioxidant, anti-inflammation, and liver-protective effects (Zhou et al., 2014). Some studies have reported that puerarin improves liver function by diminishing the accumulation of inflammatory factors and lipids in the serum (Zhou et al., 2022). Shuai Wang et al. also reported that puerarin can prevent NAFLD by maintaining mitochondrial homeostasis (Wang et al., 2019). However, the metabolic mechanism of how puerarin regulates the NAFLD model *in vivo* is still unknown. In this study, we present a novel approach for puerarin to regulate NAFLD by reducing the expression of PPARα, which is regulated by FMO5, helps protect liver cells from fatty acid-induced damage, and ultimately improves NAFLD. This study helps us understand the mechanism of action of puerarin in treating NAFLD.

## 2 Materials and methods

### 2.1 Animals

We purchased 32 C57BL/6 mice (8 weeks old) (Hangzhou Qizhen Experimental Animal Technology, Hangzhou, China) and conducted animal feeding and drug treatment at the Animal Experimental Centre of Zhejiang University of Traditional Chinese Medicine. The Institutional Animal Care and Use Committee of Zhejiang Chinese Medical University has approved the ethics of this experiment (I ACUC-20220815-15). After 1 week of adaptive feeding, the 32 mice were randomly divided into four groups: normal fat group (NFD), high-fat group (HFD), HFD + low-dose puerarin group (200 mg/kg), and HFD + high-dose puerarin group (300 mg/kg). The NFD group was fed a normal diet, and the HFD group was given a 60% high-fat diet (Research Diets, New Brunswick, United States). In addition, mice in the puerarin intervention group were gavaged 200 mg/kg/day and 300 mg/kg/day of puerarin (Solarbio, Beijing, China) after 4 weeks of high-fat feeding, respectively. The mice were weighed once a week from the start of the HFD diet. At the end of the experiments, plasma was collected under anesthesia, and relevant tissues were collected. Both the plasma and tissue samples were stored at −80°C.

### 2.2 Biochemical analysis

The plasma levels of alanine transaminase (ALT), aspartate transaminase (AST), cholesterol (TC), and TG were detected by the fully automatic animal biochemical analyzer (Mindary, Shenzhen, China). Liver malondialdehyde (MDA), superoxide dismutase (SOD), and glutathione peroxidase (GSH-px) levels were measured by MDA, SOD, and GSH-px assay kits (Beyotime Biotechnology, Shanghai, China) according to the manufacturer’s recommended protocol.

### 2.3 Histological analysis

The liver tissue was fixed in a 4% paraformaldehyde solution, embedded in paraffin, and cut into 4 μm thin sections. Hematoxylin and eosin staining (H&E) was performed on the sections to evaluate liver injury. Fresh tissue was frozen and stained with Oil red O staining solution to assess lipid deposition in the liver tissue.

### 2.4 Reverse transcription and real-time quantitative polymerase chain reaction (PCR)

Total RNA was isolated from tissues and cells using a Trizol reagent (Invitrogen, NY, United States). DS-11 (Denovix, Wilmington, DE, United States) was used to determine the content and quality of the extracted RNA, and reverse transcription was performed using HiScript III first Strand cDNA Synthesis Kit (Vazyme, Nanjing, China) according to the instructions. The real-time PCR was performed using Eva-SYBR Green Super Mix and CFX Opus 384 real-time PCR system (Bio-rad Laboratories Ltd., CA, United States). The primer sequence is shown in Supplementary Table S1.

### 2.5 Western blotting

Western blots were performed as described previously (Qinchao et al., 2023). Briefly, liver tissues or cultured cells were lysed with RIPA buffer (Applygen, Beijing, China). Centrifuged the collected RIPA lysate at 12,000 rpm at 4°C, removed the fat layer, and collected the supernatant. The BCA reagent kit (Beyotime Biotechnology, Shanghai, China) was used to quantify the protein concentration of the obtained supernatant and perform protein deformation. The protein samples were separated using 10% SDS-PAGE and transferred onto the PVDF membrane. The membrane was blocked with 5% non-fat dry milk at room temperature for 1 h. Using anti-NRF2 (Immunoway, Plano, United States), anti-PPARα (BOSTER, Wuhan, China), and anti-FMO5 (Proteintech, Wuhan, China) to hybridize target proteins. Anti-HISTONE H3 (Proteintech, Wuhan, China) and anti-β-ACTIN (Proteintech, Wuhan, China) were internal controls. Lastly, the antigen-antibody complex is detected using HRP coupled second antibody and chemiluminescence reagent.

### 2.6 AML-12 cell culture

The cells utilized in this experiment were AML-12 cells (ATCC, Manassas, VA), a type of mouse hepatocyte. AML-12 cells were cultured in DMEM/F12 - Dulbecco’s Modified Eagle Medium (Thermo Fisher Scientific, MA, United States), which was supplemented with 10% fetal bovine serum (Cellmax, Beijing, China), ITS-G (Thermo Fisher Scientific, MA, United States), 40 ng/mL dexamethasone (Sigma Aldrich, MO, United States), 100 U/mL penicillin, and 100 μg/mL streptomycin (Thermo Fisher Scientific, MA, United States). The AML-12 were incubated at 37°C in a 5% CO_2_ incubator.

### 2.7 Cell cytotoxicity assay

Cell viability was assessed using the Cell Counting Kit-8 (CCK-8) (Beyotime Biotechnology, Shanghai, China). Initially, we inoculated cells into 96-well plates when they were cultured to 80% confluence. Cells were treated with drugs and incubated for 16 h. The treated cells were tested as described in the manufacturer’s instructions. We collected the treated cell culture medium described above and used a lactate dehydrogenase (LDH) reagent kit (Thermo Fisher Scientific, MA, United States) to detect cell cytotoxicity. The propidium iodide (PI) (Thermo Fisher Scientific, MA, United States) flow cytometric assessed the loss of nuclear DNA content.

### 2.8 Lipid deposition assay

The levels of TC, TG, and non-esterified fatty acids (NEFA) in the liver were measured using the TC, TG, and NEFA determination kit (Applygen, Beijing, China). To detect the presence of intracellular lipid droplets, we used the Lipid Droplets Green Fluorescence Assay Kit with boron-dipyrromethene (BODIPY) (Beyotime Biotechnology, Shanghai, China). The treated cells were collected and washed twice with PBS. After removing the PBS, 4% paraformaldehyde fixative solution was added, and the stew was allowed at room temperature for 10–15 min. The staining solution was then added to stain the cells, and green fluorescence was observed using a fluorescence microscope.

### 2.9 Small interfering RNA (siRNA) transfection

FMO5 siRNA (siFMO5) (Santa Cruz Biotechnology, Dallas, United States) was used to knock down *FMO5* expression. When AML-12 cells reached 30%–40% confluence, siFMO5 transfection was performed using Lipofectamine™ 2000 Transfection Reagent (Thermo Fisher Scientific, MA, United States). After 24 h, palmitic acid (PA) was treated and incubated for 12 h for further analysis. Control siRNA was used as the control group in the experiment.

### 2.10 Molecular docking

The protein structures were downloaded from the AlphaFold Protein Structure Database (https://alphafold.ebi.ac.uk/) (Jumper et al., 2021; Varadi et al., 2022). The hydrogenation treatment was performed using Autodock Tools 1.5.7 software. The chemical formula of puerarin was downloaded from the PubChem database (Jumper et al., 2021). The formula was modified using ChemBioDraw 3D to generate a 3D chemical structure. The minimum energy structure was calculated, and the file was saved for subsequent docking. The docking process and binding energy recording were performed using Autodock Vina 1.1.2, and the results were visualized using PyMOL.

### 2.11 Statistical analyses

Significant differences were calculated with GraphPad Prism 5.0. Data were presented as mean ± SD values. Paired t-tests were used to analyze the statistical significance between two groups, while *F*-tests were used to analyze the statistical significance between multiple groups. *p* < 0.05 means a significant difference.

## 3 Results and discussion

### 3.1 Puerarin alleviates palmitic acid (PA)-induced *in vitro* lipotoxicity

**FIGURE 1:**
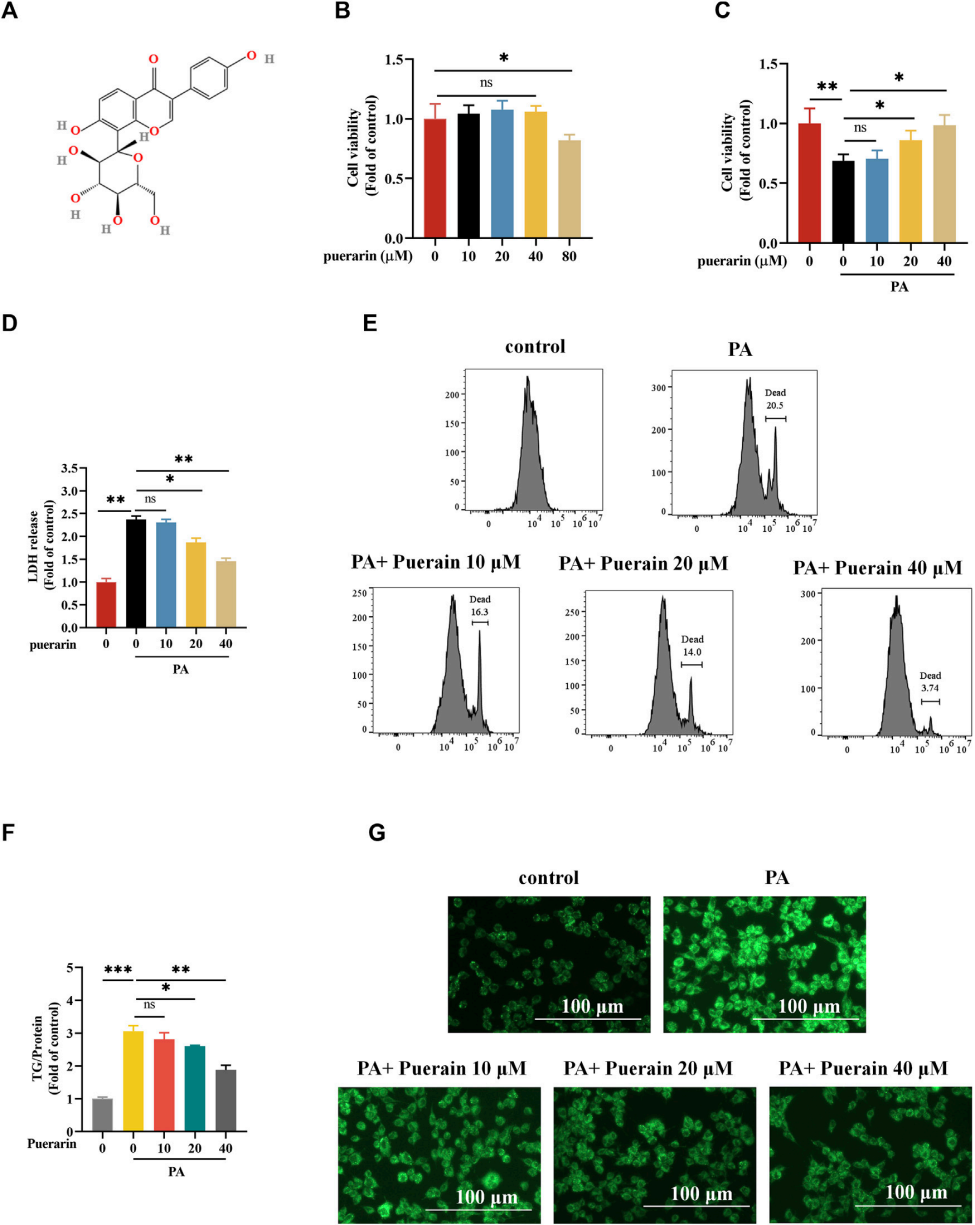
Puerarin alleviates PA-induced *in vitro* lipotoxicity. **(A)** The chemical structure of puerarin; **(B)** Cell viability after 24 h treatment with different concentrations of puerarin (0, 10, 20, 40, and 80 μM) was determined using CCK-8 assay; **(C)** AML-12 cells were exposed to PA (500 μM) for 12 h and puerarin (0, 10, 20, and 40 μM) was added 2 h before PA (500 μM) treatment and cell viability was determined using CCK-8 assay. **(D–E)** Cell death was detected by measurements of LDH release and PI staining; **(F)** Intracellular TG content after exposure to puerarin (0, 10, 20, and 40 μM) and PA (500 μM); **(G)** Representative Bodipy staining of AML-12 cell. * means *p* < 0.05, ** means *p* < 0.01, ***means *p* < 0.001.

The chemical structure of puerarin is shown in Figure 1A. We first evaluated the safe dose of puerarin to hepatocytes using the CCK8 assay. No cytotoxicity was induced in AML-12 hepatocytes at doses of puerarin less than or equal to 40 μM (Figure 1B). Furthermore, we determined the protective effect of puerarin on PA-induced AML-12 hepatocyte damage by CCK8, LDH, and PI staining. The results show that puerarin significantly ameliorated PA-induced hepatocyte injury in a gradient-dependent manner (Figures 1C–E). PA induces hepatocyte damage and significantly increases hepatocyte lipid deposition (Yang et al., 2020). To clarify whether puerarin ameliorated PA-induced lipid deposition, we evaluated it by intracellular triglyceride assay and Bodipy staining. Our data show that PA-induced lipid deposition in AML-12 hepatocytes was attenuated by puerarin in a dose-dependent manner (Figure 1F,G). These results suggest that puerarin protects hepatocytes from palmitate-induced injury and lipid deposition.

### 3.2 Puerarin attenuates high-fat diet-induced liver injury

**FIGURE 2:**
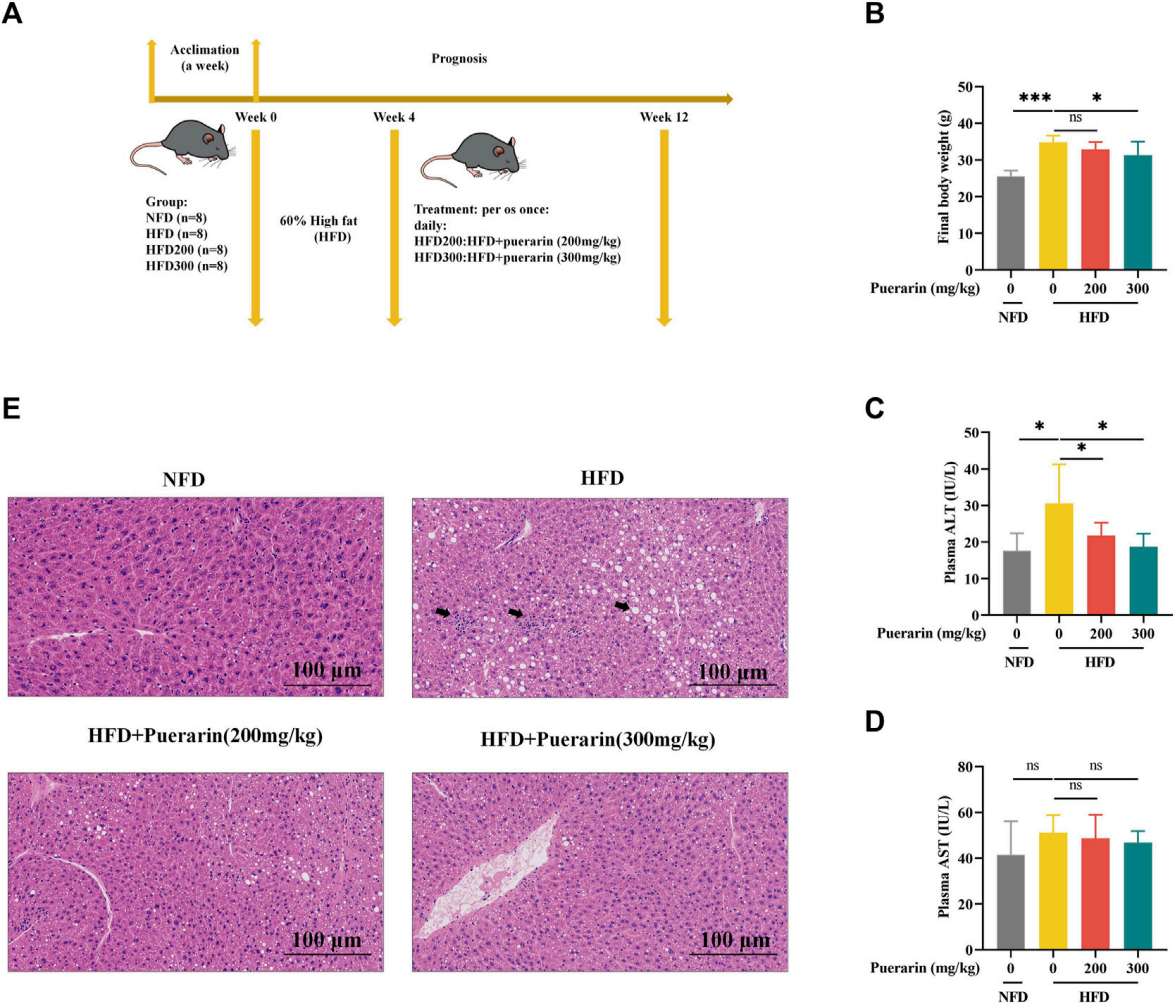
Puerarin attenuates high-fat diet-induced liver injury. **(A)** Schematic diagram of animal feeding; **(B)** Final mouse body weight; **(C)** plasma ALT; **(D)** plasma AST; **(E)** Representative H&E staining of the liver. The scale bar length represents 100 μm * means *p* < 0.05, ** means *p* < 0.01, ***means *p* < 0.001. NFD, normal fat diet; HFD, high-fat diet; HFD200, high-fat diet with puerarin (200 mg/kg/day) intervention; HFD300, high-fat diet with puerarin (300 mg/kg/day) intervention.

To clarify the effect of puerarin on NAFLD, we established an NAFLD model using a high-fat diet and intervened with puerarin after 4 weeks. The feeding pattern is shown in Figure 2A. During feeding, the mobility of HFD mice was significantly decreased compared with NFD, while their mobility was enhanced in the puerarin intervention group; moreover, the intervention of puerarin significantly inhibited HFD-induced obesity in mice (Figure 2B). ALT and AST levels are important indicators for assessing liver injury, and our results showed that puerarin treatment significantly inhibited the HFD-induced increase in plasma ALT levels, but there was no difference in AST levels (Figure 2C,D). We observed pathophysiological changes in mouse livers via H&E staining (Figure 2E) and found that treatment with puerarin reversed HFD-induced hepatic pathophysiological changes. This indicated that puerarin intervention can significantly improve liver damage induced by a high-fat diet.

### 3.3 Puerarin ameliorates high-fat diet-induced hepatic lipid deposition

**FIGURE 3:**
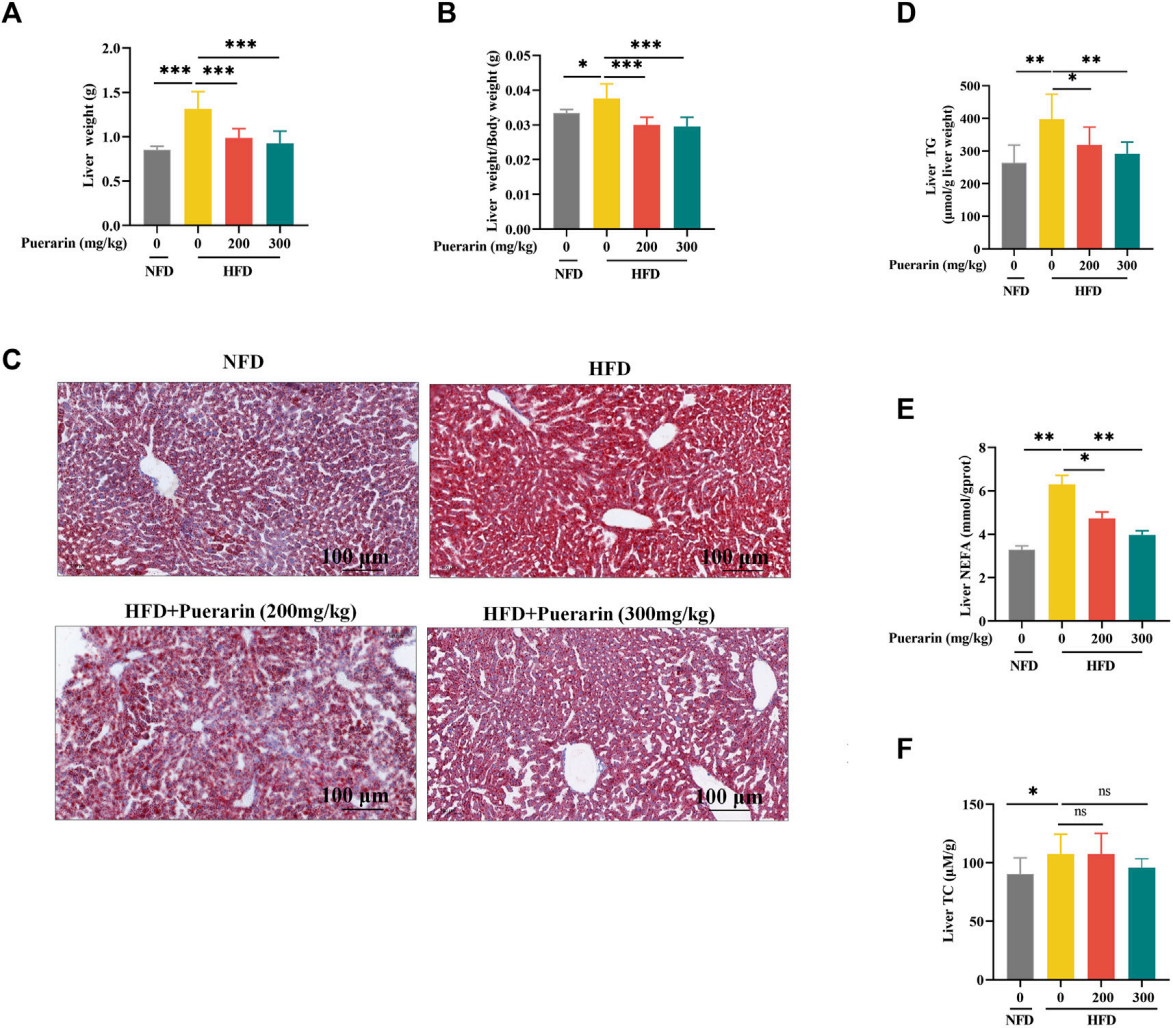
Puerarin ameliorates high-fat diet-induced hepatic lipid deposition. **(A)** Liver weight; **(B)** The liver-to-body-weight ratio of mice; **(C)** Representative Oil Red O staining of the liver; **(D)** TG levels in the liver; **(E)** NFFA levels in the liver; **(F)** TC levels in the liver. The scale bar length represents 100 μm * means *p* < 0.05, ** means *p* < 0.01, ***means *p* < 0.001. NFD, normal fat diet; HFD, high-fat diet; HFD200, high-fat diet with puerarin (200 mg/kg/day) intervention; HFD300, high-fat diet with puerarin (300 mg/kg/day) intervention.

In our study, we evaluated the impact of puerarin on hepatic lipid deposition in mice. The HFD group significantly exacerbated the liver weight of mice fed a high-fat diet compared with the NFD group, and the puerarin intervention significantly restored the changes in liver weight (Figure 3A,B). Compared with NFD, a significant increase in lipid droplet vacuoles in the liver was observed in the HFD group by oil red O staining (Figure 3C). Fewer lipid droplet vacuoles were observed after puerarin treatment. Meanwhile, to further evaluate puerarin’s effect on HFD-induced hepatic steatosis, the levels of TG and NEFA in the liver were monitored. The results showed a sharp increase in hepatic TG and NEFA levels in the HFD group compared to the NFD group (Figure 3D,E). Puerarin treatment significantly reduced hepatic TG and NEFA levels, with almost complete recovery in the high-dose puerarin group. However, puerarin treatment did not significantly affect the high-fat-induced increase in TC (Figure 3F).

### 3.4 Puerarin restores antioxidant function impaired by high-fat diets

**FIGURE 4:**
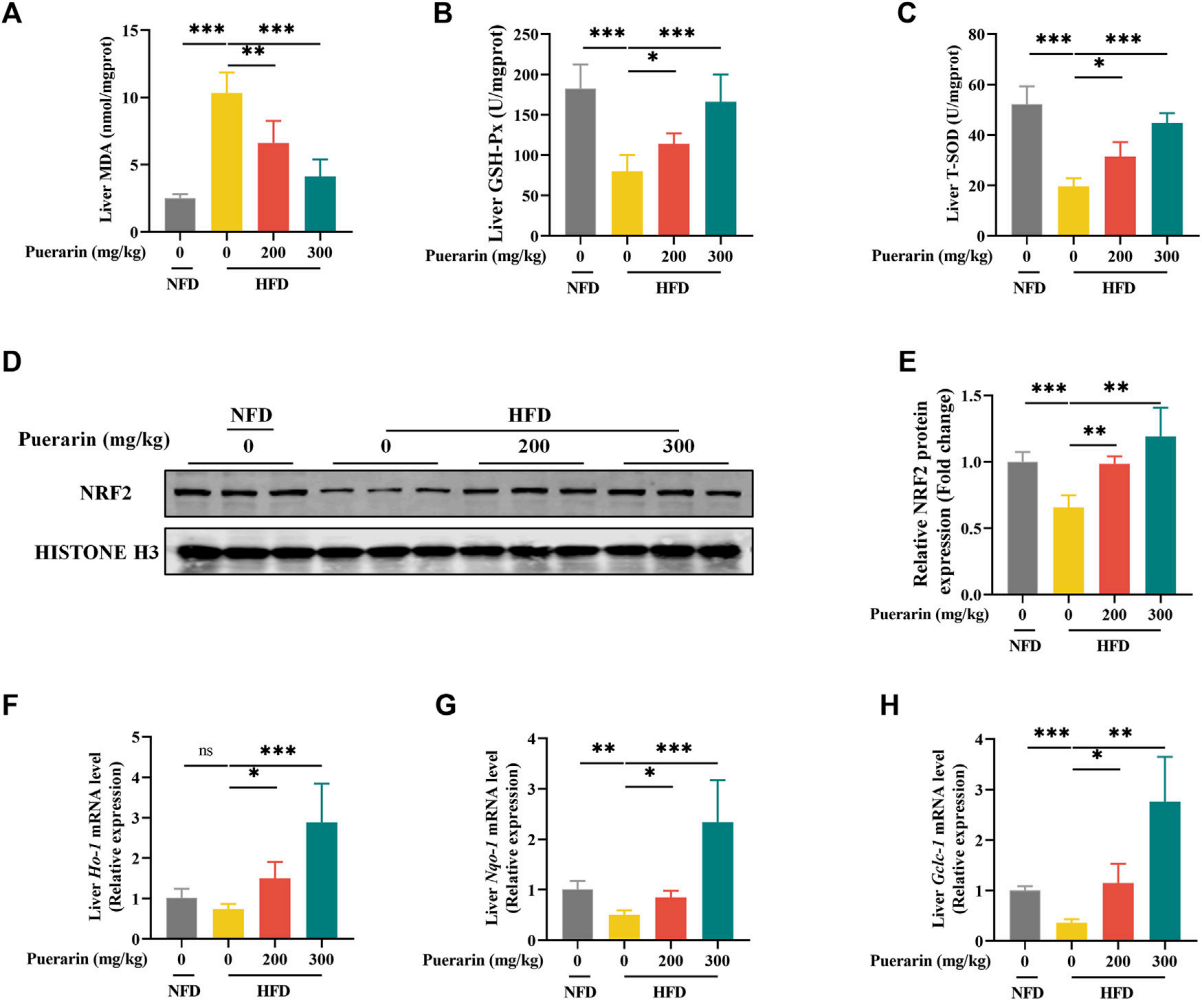
Puerarin restores antioxidant function impaired by high-fat diets. **(A)** MDA content in the liver; **(B)** GSH-Px in the liver; **(C)** T-SOD activity in the liver; **(D)** Nrf2 protein expression was detected by Western blotting; **(F)** Nrf2 quantification with ImageJ; **(E–H)** qRT-PCR showing the expressions of *Ho-1*, *Nqo-1*, and *Gclc-1* in each group of mouse livers. * means *p* < 0.05, ** means *p* < 0.01, ***means *p* < 0.001. NFD, normal fat diet; HFD, high-fat diet; HFD200, high-fat diet with puerarin (200 mg/kg/day) intervention; HFD300, high-fat diet with puerarin (300 mg/kg/day) intervention.

Lipid peroxidation, caused by excessive lipid accumulation in the liver, is known to play a central role in the development and progression of NAFLD (Tan and Norhaizan, 2019). Producing lipid peroxidation products such as MDA attacks hepatocytes, damaging hepatocytes (Witayavanitkul et al., 2020). Therefore, we prioritized the measurement of hepatic MDA content and showed that the HFD group significantly increased the level of MDA compared to the NFD group, which was reversed by puerarin (Figure 4A). Furthermore, we investigated whether the puerarin intervention had a modulatory effect on high-fat-induced oxidative stress. We measured the levels of SOD and GSH-px in mice liver, which are commonly used markers of oxidative stress. The results showed that puerarin intervention significantly restored the high-fat diet-induced enzyme activities of hepatic SOD and GSH-px in mice liver (Figures 4A–C), indicating that puerarin may help counteract oxidative stress induced by a high-fat diet.

Nuclear factor erythroid-2-related factor 2 (NRF2) is vital in reducing cell oxidative stress by regulating various mechanisms (Ma, 2013; Loboda et al., 2016). NRF2 translocation to the nucleus induces the transcriptional expression of antioxidant genes, and our results showed that HFD significantly decreased the protein expression level of NRF2 in the nucleus, whereas puerarin significantly increased the expression of NRF2 (Figure 4D,E). To verify that NRF2 exerts a transcriptional function, we examined the transcriptionally expressed genes downstream of *Nrf2*, and our results showed that puerarin significantly activated the expression of *Ho-1*, *Nqo-1,* and *Gclc-1* genes downstream of *Nrf2* (Figures 4F–H). The results suggest that puerarin significantly restores the body’s antioxidant function and is associated with the NRF2 signaling pathway.

### 3.5 FMO5/PPAR*α* signaling pathway is involved in puerarin-regulated lipid metabolism

**FIGURE 5:**
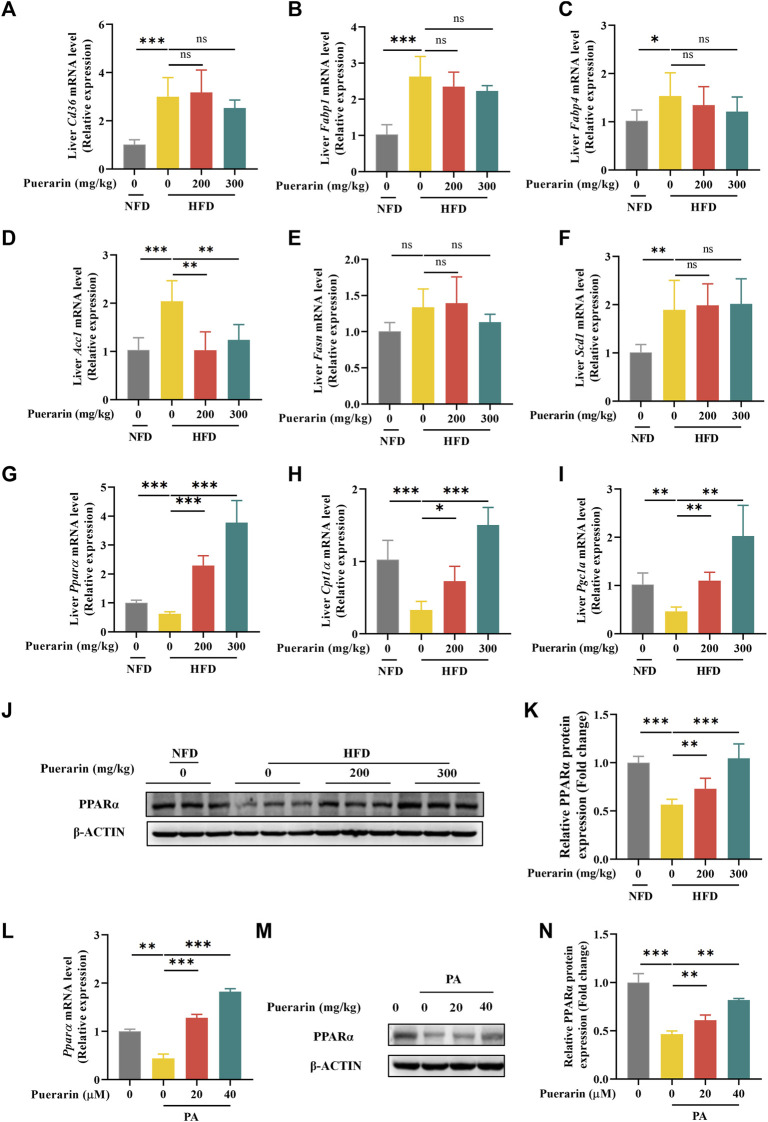
Puerarin regulates hepatic fatty acid metabolism. **(A–I)** qRT-PCR detected the expressions of *Cd36*, *Fabp1*, *Fabp4*, *Acc1*, *Fasn*, *Scd1*, *Ppar-α*, *Cpt1α*, *and Pgc1α* in each group of mouse livers. **(J–K)** The expression of PPARα in mouse liver was detected by Western blotting and q-PCR. **(L–N)** The expression of PPARα in AML-12 cells treated with puerarin (0, 20, and 40 μM) and PA (500 μM) was detected by Western blotting and q-PCR. * means *p* < 0.05, ** means *p* < 0.01, ***means *p* < 0.001. NFD, normal fat diet; HFD, high-fat diet; HFD200, high-fat diet with puerarin (200 mg/kg/day) intervention; HFD300, high-fat diet with puerarin (300 mg/kg/day) intervention.

Chronic high-fat diet intake results in a marked increase in the expression of proteins involved in fatty acid uptake and fatty acid synthesis, and mitochondrial dysfunction contributes to diminished fatty acid β-oxidation (Badmus et al., 2022). To investigate the regulation of lipid metabolism by puerarin, we measured gene expression for fatty acid uptake (*Cd36*, *Fabp1*, and *Fabp4*), synthesis (*Acc1*, *Fasn*, and *Scd1*), and β-oxidation (*Cpt1α*, *Ppar-α*, and *Pgc1α*). Consistent with existing reports, genes regulated by fatty acid β-oxidation were significantly repressed, and the treatment with puerarin significantly reversed these phenomena. However, we did not observe an increase in the expression of genes involved in fatty acid uptake and synthesis in the HFD group by puerarin (Figures 5A–I). In addition, we observed that *Ppar-α* expression was highly activated in puerarin-regulated lipid metabolism genes, and we verified at the protein level that puerarin significantly activated PPARα expression in the liver (Figure 5J,K). Furthermore, the *in vitro* results were consistent with the *in vivo* results (Figure 5L–N).

**FIGURE 6:**
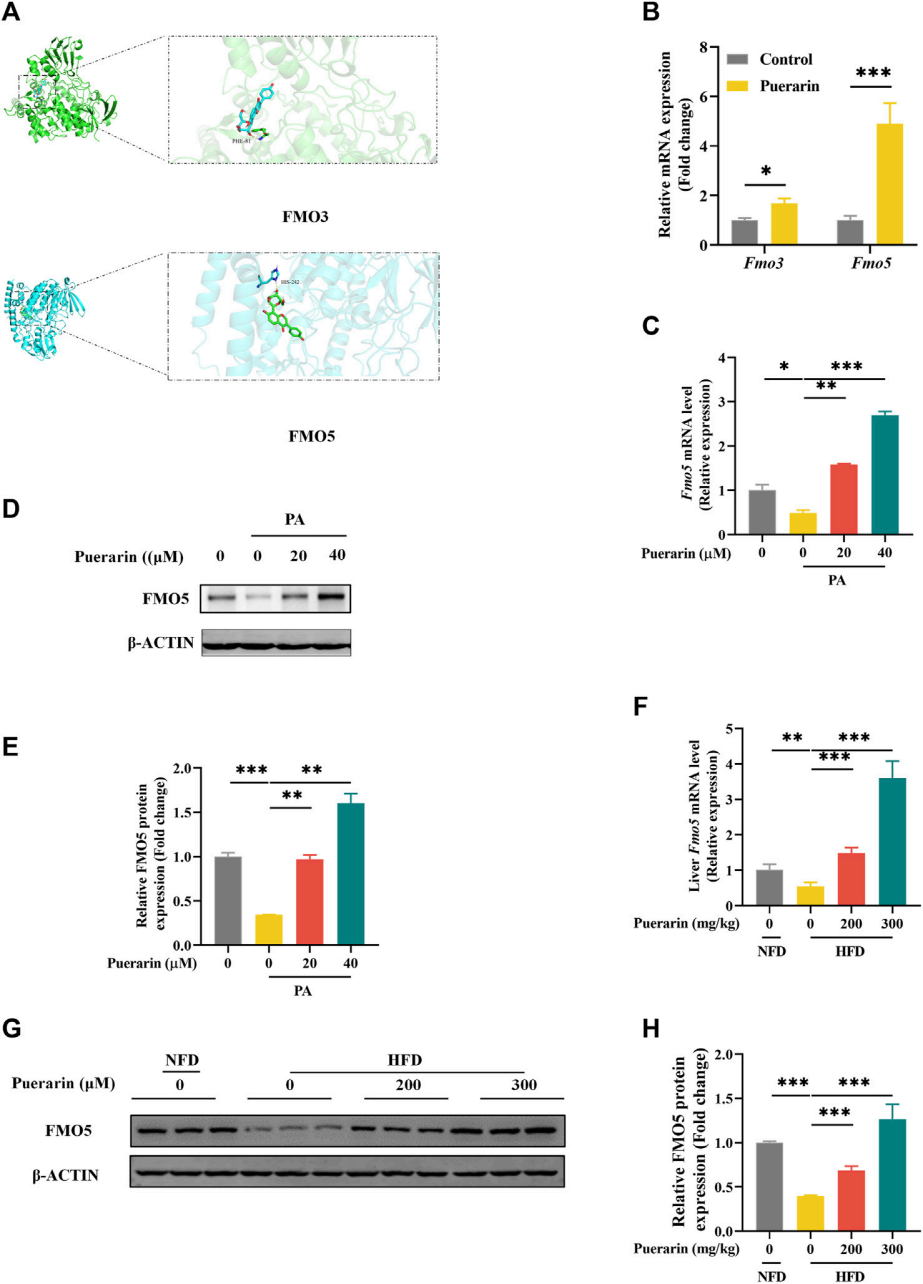
FMO5/PPAR-α signaling pathway is involved in puerarin-regulated lipid metabolism. **(A)** Molecular interaction of puerarin in the catalytic site of FMO3/FMO5; **(B)** qRT-PCR detected the expressions of *Fmo3/Fmo5* in each group of mouse livers; **(C–E)** The expression of FMO5 in AML-12 cells which were treated by puerarin (0, 20, and 40 μM) and PA (500 μM) was detected by Western blotting and q-PCR. **(F)** qRT-PCR and Western blotting detected the expressions of FMO5 in mouse liver. **(G–H)** FMO5 protein expression in mouse liver was detected by Western blotting and quantification with ImageJ. * means *p* < 0.05, ** means *p* < 0.01, ***means *p* < 0.001. NFD, normal fat diet; HFD, high-fat diet; HFD200, high-fat diet with puerarin (200 mg/kg/day) intervention; HFD300, high-fat diet with puerarin (300 mg/kg/day) intervention.

To further explore the key factors in the modulation of lipid metabolism by puerarin, we speculated that it might be related to the drug metabolism of puerarin. FMOs are involved in the metabolism of small-molecule drugs. In humans, FMO3 and FMO5 are involved in drug metabolism in the liver (Reddy et al., 2010). Molecular docking was used to reveal the interaction relationship between protein and drug. The results of the docking analysis displayed that the binding energy of docked FMO3 and puerarin was −8.6 kcal/mol, and the binding energy of docked FMO5 and puerarin was −9.4 kcal/mol (Figure 6A). We validated this in AML-12 cells and showed that puerarin treatment alone significantly activated the expression of *Fmo3* and *Fmo5*, and in comparison, *Fmo5* was more significantly regulated by puerarin (Figure 6B). In addition, puerarin significantly restored the gene and protein expression of FMO5, which was significantly downregulated by PA (Figures 6C–E), and similarly, puerarin reversed the inhibition of FMO5 expression by HFD (Figures 6F–H). These results suggest that puerarin-regulated lipid metabolism is associated with the FMO5/PPARα signaling pathway.

### 3.6 Block of FMO5 inhibits hepatic lipotoxicity protection by puerarin

**FIGURE 7:**
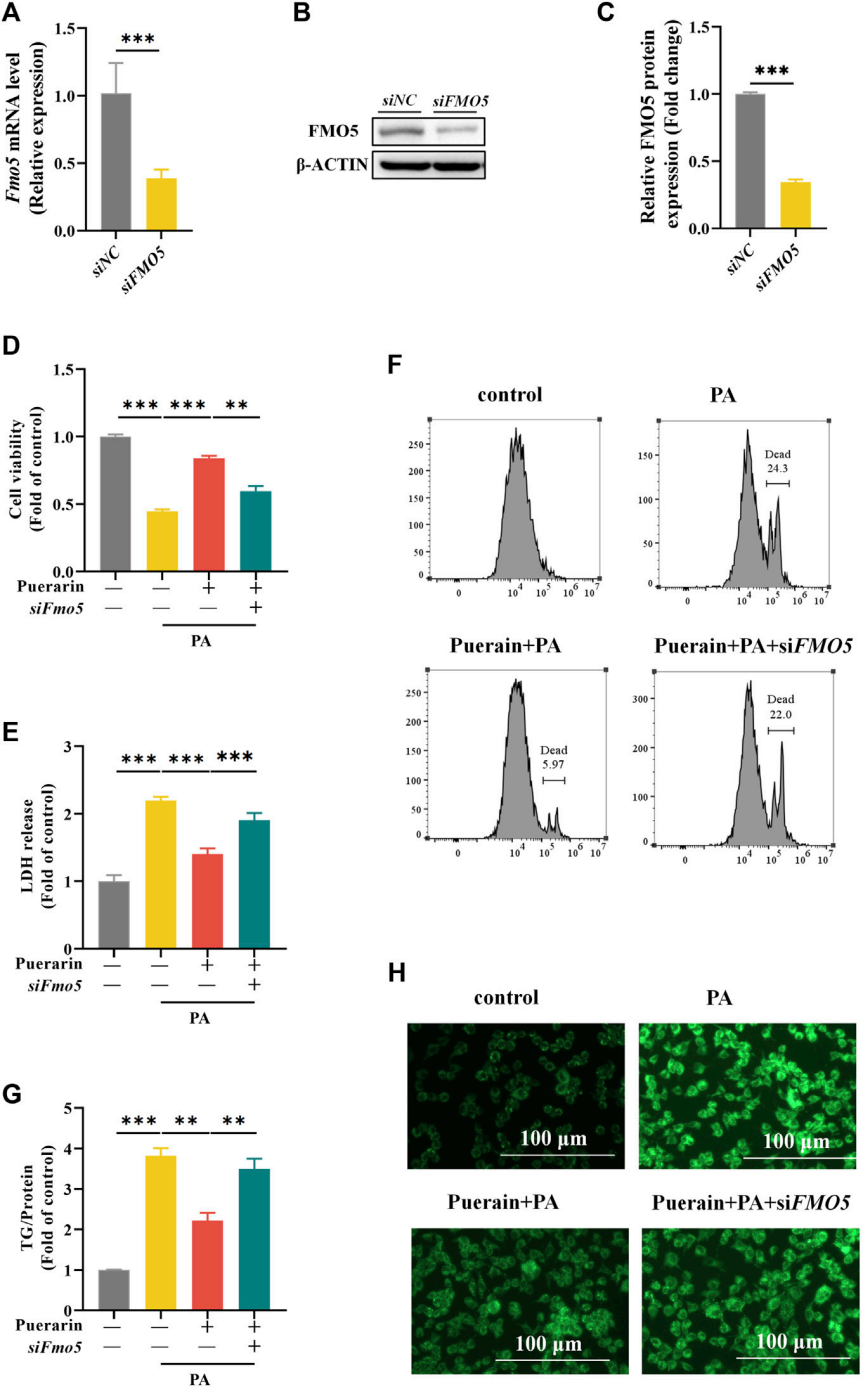
Inhibit FMO5 blocks the effect of puerarin on PA-induced hepatocyte viability and lipid deposition. **(A–C)** Western blot and RT-PCR assays were used to detect the protein and mRNA levels of FMO5 in *si*Fmo5 transfected AML-12 cells. **(D–F)** Cck-8, LDH and PI staining assays were used to detect the cell damage after AML-12 cells were treated with *si*Fmo5, puerarin (40 μM), and PA (500 μM). **(G,H)** TG and Bodipy assays were used to detect the lipid deposition after AML-12 cells were treated with siFMO5, puerarin (40 μM), and PA (500 μM). * means *p* < 0.05, ** means *p* < 0.01, ***means *p* < 0.001.

To further validate the role of FMO5 in protecting puerarin against hepatic lipotoxicity, we silenced the expression of *Fmo5* using siRNA transfection of *Fmo5* with knockdown efficiency, as shown in Figures 7A–C. Meanwhile, we found that inhibition of FMO5 significantly inhibited PPARα (Supplementary Figure S2). The results showed that FMO5 knockdown significantly inhibited the protective effect of puerarin on PA-induced lipid deposition in AML-12 hepatocytes (Figures 7D–F). In addition, FMO5 inhibition also reversed the protective effect of puerarin on PA-mediated AML-12 hepatocyte injury (Figure 7G,H). These results demonstrate that FMO5 is mechanistically involved in the protective role of puerarin against PA-induced hepatic lipotoxicity.

**FIGURE 8:**
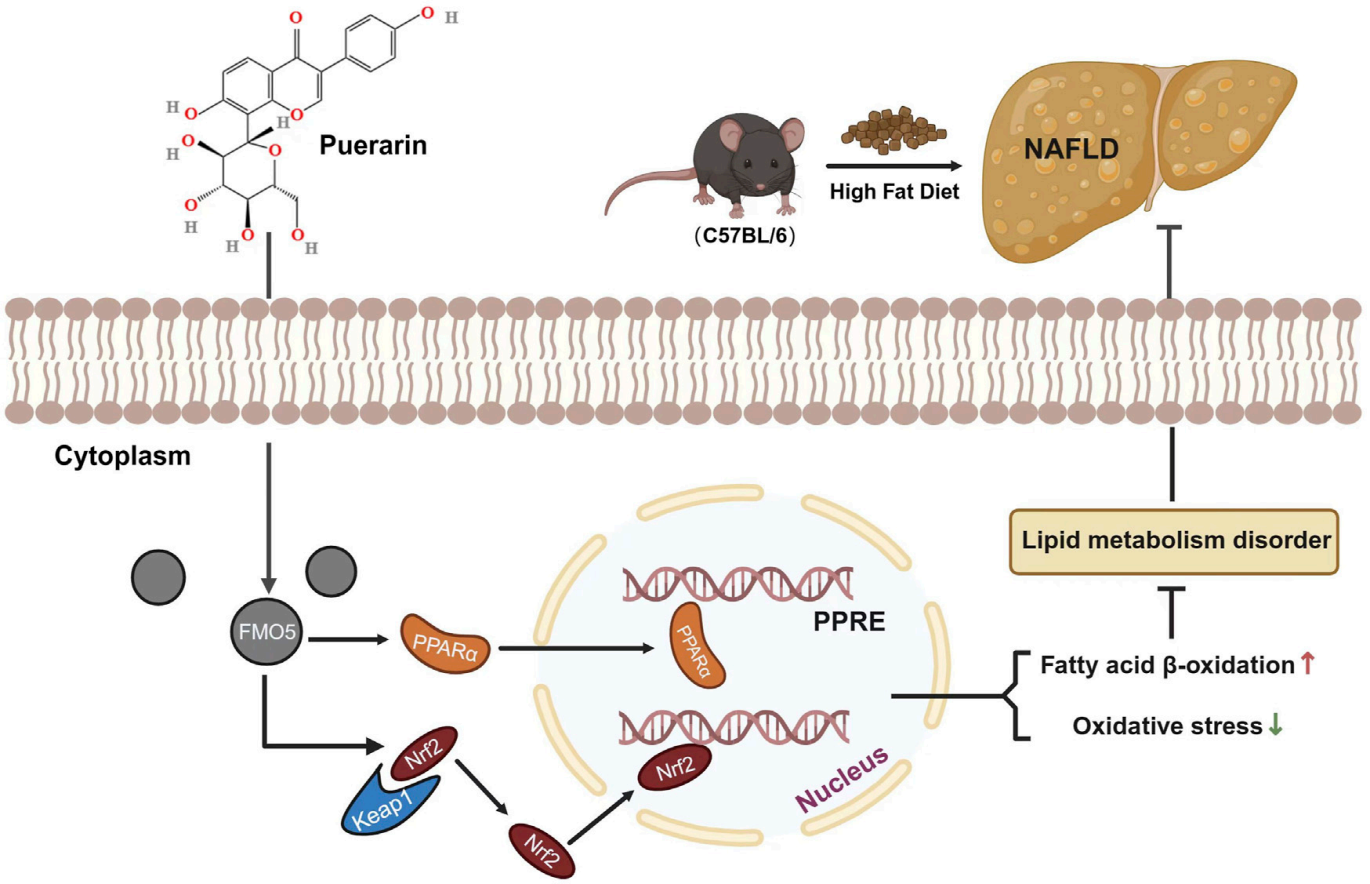
Illustration of puerarin interaction with FMO5 for protection against NAFLD. Puerarin increased the expression of NRF2 in the nucleus. This action upregulated the expression of antioxidant stress-related genes and inhibited the increase in MDA caused by NAFLD. It also restored the activity of SOD and GSH-px, reduced NAFLD-induced oxidative stress, and ameliorated liver injury. Additionally, FMO5 could regulate the PPARα signaling pathway and activate various fatty acid oxidase genes, thereby reducing lipid accumulation in the liver.

## 4 Discussion

Our research has shown that puerarin can protect against HFD-induced liver injury and lipid accumulation in mice. Puerarin was also found to reduce oxidative stress levels in the liver significantly. Furthermore, both *in vitro* and *in vivo* experiments have demonstrated that puerarin can protect hepatocytes from HFD-induced damage, and potential mechanisms were modulation of the FMO5/PPARα signaling pathway.

Puerarin is an extract of *Puraria lobate*, a plant with medicinal and food applications. It is widely used in industries such as pharmaceuticals, food, and health products, making it a promising market contender (Xuan et al., 2023). Although it is believed that *Puraria lobate* can resist oxidation and improve blood lipids, the reasons for this phenomenon remain unclear. Previous studies have shown that *Puraria lobata* can enhance the intestinal microenvironment by increasing the abundance of *lactobacillus*, *bifidobacterium*, and *tubriciactors*, thereby ameliorating NAFLD inflammation, hepatic steatosis, and elevated blood lipids induced by high-fat and high-cholesterol diet through the liver-gut axis (Yang et al., 2020). Research by Pattawika Lertpatipanpong et al. shows that isoflavones in *Pueraria lobata* have pharmacological activity and beneficial effects on NAFLD, hyperglycemia, and hyperlipidemia (Lertpatipanpong et al., 2020). Puerarin, the primary flavonoid derivative extract of *Pueraria lobata*, has been shown to ameliorate high-fat diet-induced hyperlipidemia by increasing skeletal muscle energy metabolism (Guerra et al., 2000; Jung et al., 2017). Based on previous findings, we conducted a study showing biochemical and pathological results that after intervention with puerarin, the lipid droplets in the liver of mice decreased significantly, and the area of injury was significantly reduced. These results indicate that puerarin significantly improved NAFLD-induced liver injury and lipid accumulation, suggesting its potential as a drug for treating NAFLD. However, surprisingly, puerarin intervention did not improve cholesterol levels in the liver of mice. This indicates that in addition to regulating traditional lipid metabolism pathways, puerarin may have unknown regulatory pathways to improve NAFLD.

Accumulating evidence suggests that oxidative stress plays a central role in NAFLD (Kang et al., 2022). Excessive oxidative stress can promote the progression of NAFLD, and the marker is converted to non-alcoholic steatohepatitis, which promotes liver fibrosis (Zou et al., 2021). MDA is a lipid peroxidation product and is associated with oxidative stress. Our data show that puerarin intervention can significantly inhibit the increase in MDA induced by hyperlipidemia. Puerarin can also restore SOD and GSH-px activity, which is a key defense system against oxidative stress. NRF2 is a key transcription factor maintaining intracellular redox homeostasis (Anuranjani, 2014). NAFLD reduces NRF2 phosphorylation, prevents NRF2 nuclear translocation, and inhibits downstream antioxidant stress-related enzymes (Kang et al., 2022), including HO-1 and NQO-1. In our study, puerarin increased NRF2 protein expression while activating its downstream proteins HO-1 and NQO-1.

Abnormal lipid metabolism is another important indicator of NAFLD progression. The liver, the central organ of lipid metabolism, is exposed to a large amount of free fatty acids (FFA) and TG environment under high-fat feeding (Rada et al., 2020). The excessive accumulation of lipids and their derivatives, particularly saturated fatty acids in the liver, can trigger cellular stress responses, leading to the progression of NAFLD (Mota et al., 2016; Heeren and Scheja, 2021). Therefore, we apply PA to imitate lipotoxicity *in vitro*. FFA, especially saturated fatty acids, enter the cell through transmembrane proteins such as CD36, FABP1, and FABP4 (Rada et al., 2020; Yu et al., 2021; Lv et al., 2023). During fasting or exercise, fatty acids undergo mitochondria β oxidation to provide the essential metabolic fluxes in the catalysis of CPT1 and PGC1α (Schreurs et al., 2010; Rui, 2014). Nevertheless, in the presence of chronic energy excess, common in NAFLD, fatty acids continuously generate TG under the action of enzymes (Heeren and Scheja, 2021). Acetyl-CoA is a crucial intermediate in this process. The enzyme ACC1 converts acetyl CoA into malonyl CoA, while FASN and SCD1 convert malonyl CoA into monounsaturated fatty acids. These monounsaturated fatty acids then undergo glycerol esterification and are removed by low-density lipoprotein (Iizuka et al., 2020). If the synthesis of triglycerides exceeds the storage capacity of liver cells and simultaneously transcends the secretion of low-density lipoprotein, liver steatosis will occur (Heeren and Scheja, 2021). We did not see any improvement in puerarin on *Fasn* and *Scd1* except for *Acc1*. However, we found that puerarin activated *Cpt1α* and *Pgc1α*. In addition, we also observe that puerarin intervention can upregulate the transcription levels of *Pparα*, which is a nuclear hormone receptor, can activate various fatty acid oxidase enzymes such as CPT1 and PGC1α and regulate lipid metabolism and inflammation (Wu et al., 2019; Liu et al., 2020; Yang et al., 2023). Therefore, we focus on how puerarin can improve NAFLD via PPARα.

According to Lingjian Kong et al., PPARα interacts with FMO5, and inhibiting FMO5 increases liver damage and lipid accumulation (Kong et al., 2021). Reviews report the effects of FMO5 in altering metabolism and energy balance and participating in Nrf2-mediated oxidative stress response and lipid balance, which are profoundly affected in NAFLD (Rossner et al., 2017; Phillips et al., 2023). Consequently, we further investigated the changes in FMO5 in NAFLD treated with puerarin. We found that puerarin effectively improved the induction of FMO5 reduction in HFD, consistent with the results of *in vitro* assays. Additionally, FMO5 can regulate PPARα and plays a crucial role in improving saturated fatty acid-induced liver cell apoptosis and lipid accumulation by puerarin. Notably, enhanced FMO5 expression can protect against PA-induced liver cell damage, indicating that FMO5 is involved in the mechanism of its beneficial role in protecting against NAFLD.

Our study established an NAFLD mouse model by feeding C57BL/6 mice a high-fat diet. We discovered that puerarin could alleviate high-fat-diet-induced liver injury and lipid accumulation through the FMO5/PPAR-α signaling pathway. However, our research does have limitations. We only established a NAFLD model using high-fat feed. Although this model is well-described and mimics the features of human disease, including obesity, insulin resistance, and hyperlipidemia (Tetri et al., 2008), it induces less severe steatosis and inflammation compared to a methionine and choline-deficient diet (Van Herck et al., 2017). This model also does not commonly cause liver cancers. It is important to note that there are inherent differences between mice and humans, such as in the distribution of adipose tissue, which cannot be altered when using rodent models. Moreover, due to the complex and multi-directional nature of NAFLD pathophysiology, no perfect animal model fully represents the complete NAFLD spectrum.

Overall, our research suggests that puerarin may be a potential therapeutic drug for treating high-fat-diet-induced obesity and lipid deposition. Additionally, we found that inhibiting FMO5 can inhibit PPARα, and we speculate that FMO5 may work synergistically with PPARα to improve NAFLD. This may provide new ideas for designing therapeutic drugs targeting PPARα.

## 5 Conclusion

This study aimed to investigate how intervention with puerarin can ameliorate liver cell damage and lipid accumulation induced by a high-fat diet. We found that puerarin works by regulating the FMO5/PPARα signaling pathway, providing a promising experimental basis for the potential therapeutic application of puerarin in preventing NAFLD.

## Data Availability

The raw data supporting the conclusions of this article will be made available by the authors, without undue reservation.

## References

[B1] Anuranjani B. M. (2014). Concerted action of Nrf2-ARE pathway, MRN complex, HMGB1 and inflammatory cytokines - implication in modification of radiation damage. Redox Biol. 2, 832–846. 10.1016/j.redox.2014.02.008 25009785 PMC4085347

[B2] Badmus O. O. Hillhouse S. A. Anderson C. D. Hinds T. D. Stec D. E. (2022). Molecular mechanisms of metabolic associated fatty liver disease (MAFLD): functional analysis of lipid metabolism pathways. Clin. Sci. (Lond) 136 (18), 1347–1366. 10.1042/CS20220572 36148775 PMC9508552

[B3] Guerra M. C. Speroni E. Broccoli M. Cangini M. Pasini P. Minghett A. (2000). Comparison between Chinese medical herb Pueraria lobata crude extract and its main isoflavone puerarin antioxidant properties and effects on rat liver CYP-catalysed drug metabolism. Life Sci. 67 (24), 2997–3006. 10.1016/s0024-3205(00)00885-7 11133012

[B4] Heeren J. Scheja L. (2021). Metabolic-associated fatty liver disease and lipoprotein metabolism. Mol. Metab. 50, 101238. 10.1016/j.molmet.2021.101238 33892169 PMC8324684

[B5] Iizuka K. Takao K. Yabe D. (2020). ChREBP-mediated regulation of lipid metabolism: involvement of the gut microbiota, liver, and adipose tissue. Front. Endocrinol. (Lausanne) 11, 587189. 10.3389/fendo.2020.587189 33343508 PMC7744659

[B6] Ipsen D. H. Lykkesfeldt J. Tveden-Nyborg P. (2018). Molecular mechanisms of hepatic lipid accumulation in non-alcoholic fatty liver disease. Cell Mol. Life Sci. 75 (18), 3313–3327. 10.1007/s00018-018-2860-6 29936596 PMC6105174

[B7] Jumper J. Evans R. Pritzel A. Green T. Figurnov M. Ronneberger O. (2021). Highly accurate protein structure prediction with AlphaFold. Nature 596 (7873), 583–589. 10.1038/s41586-021-03819-2 34265844 PMC8371605

[B8] Jung H. W. Kang A. N. Kang S. Y. Park Y. K. Song M. Y. (2017). The root extract of Pueraria lobata and its main compound, puerarin, prevent obesity by increasing the energy metabolism in skeletal muscle. Nutrients 9 (1), 33. 10.3390/nu9010033 28054981 PMC5295077

[B9] Kang Y. Song Y. Luo Y. Song J. Li C. Yang S. (2022). Exosomes derived from human umbilical cord mesenchymal stem cells ameliorate experimental non-alcoholic steatohepatitis via Nrf2/NQO-1 pathway. Free Radic. Biol. Med. 192, 25–36. 10.1016/j.freeradbiomed.2022.08.037 36096356

[B10] Kong L. Chen J. Ji X. Qin Q. Yang H. Liu D. (2021). Alcoholic fatty liver disease inhibited the co-expression of Fmo5 and PPARα to activate the NF-κB signaling pathway, thereby reducing liver injury via inducing gut microbiota disturbance. J. Exp. Clin. Cancer Res. 40 (1), 18. 10.1186/s13046-020-01782-w 33413501 PMC7788704

[B11] Lertpatipanpong P. Janpaijit S. Park E. Y. Kim C. T. Baek S. J. (2020). Potential anti-diabetic activity of Pueraria lobata flower (flos puerariae) extracts. Molecules 25 (17), 3970. 10.3390/molecules25173970 32878147 PMC7504745

[B12] Liu Y. Sun L. Zheng L. Su M. Liu H. Wei Y. (2020). Spexin protects cardiomyocytes from hypoxia-induced metabolic and mitochondrial dysfunction. Naunyn Schmiedeb. Arch. Pharmacol. 393 (1), 25–33. 10.1007/s00210-019-01708-0 31396649

[B13] Loboda A. Damulewicz M. Pyza E. Jozkowicz A. Dulak J. (2016). Role of Nrf2/HO-1 system in development, oxidative stress response and diseases: an evolutionarily conserved mechanism. Cell Mol. Life Sci. 73 (17), 3221–3247. 10.1007/s00018-016-2223-0 27100828 PMC4967105

[B14] Loomba R. Friedman S. L. Shulman G. I. (2021). Mechanisms and disease consequences of nonalcoholic fatty liver disease. Cell 184 (10), 2537–2564. 10.1016/j.cell.2021.04.015 33989548 PMC12168897

[B15] Lv J. Hu Y. Li L. He Y. Wang J. Guo N. (2023). Targeting FABP4 in elderly mice rejuvenates liver metabolism and ameliorates aging-associated metabolic disorders. Metabolism 142, 155528. 10.1016/j.metabol.2023.155528 36842611

[B16] Ma Q. (2013). Role of nrf2 in oxidative stress and toxicity. Annu. Rev. Pharmacol. Toxicol. 53, 401–426. 10.1146/annurev-pharmtox-011112-140320 23294312 PMC4680839

[B17] Mota M. Banini B. A. Cazanave S. C. Sanyal A. J. (2016). Molecular mechanisms of lipotoxicity and glucotoxicity in nonalcoholic fatty liver disease. Metabolism 65 (8), 1049–1061. 10.1016/j.metabol.2016.02.014 26997538 PMC4931958

[B18] Paternostro R. Trauner M. (2022). Current treatment of non-alcoholic fatty liver disease. J. Intern Med. 292 (2), 190–204. 10.1111/joim.13531 35796150 PMC9546342

[B19] Phillips I. R. Veeravalli S. Khadayate S. Shephard E. A. (2023). Metabolomic and transcriptomic analyses of Fmo5-/- mice reveal roles for flavin-containing monooxygenase 5 (FMO5) in NRF2-mediated oxidative stress response, unfolded protein response, lipid homeostasis, and carbohydrate and one-carbon metabolism. PLoS One 18 (6), e0286692. 10.1371/journal.pone.0286692 37267233 PMC10237457

[B20] Powell E. E. Wong V. W. Rinella M. (2021). Non-alcoholic fatty liver disease. Lancet 397 (10290), 2212–2224. 10.1016/S0140-6736(20)32511-3 33894145

[B21] Qinchao D. R. G. Liuyi H. Song Q. Ai F. Shanglei L. Tiantian X. (2023). Hepatic TRPC3 loss contributes to chronic alcohol consumption-induced hepatic steatosis and liver injury in mice. Life Metab. 3 (1). 10.1093/lifemeta/load050 PMC11749259 39871879

[B22] Rada P. Gonzalez-Rodriguez A. Garcia-Monzon C. Valverde Á. M. (2020). Understanding lipotoxicity in NAFLD pathogenesis: is CD36 a key driver? Cell Death Dis. 11 (9), 802. 10.1038/s41419-020-03003-w 32978374 PMC7519685

[B23] Reddy R. R. Ralph E. C. Motika M. S. Zhang J. Cashman J. R. (2010). Characterization of human flavin-containing monooxygenase (FMO) 3 and FMO5 expressed as maltose-binding protein fusions. Drug Metab. Dispos. 38 (12), 2239–2245. 10.1124/dmd.110.033639 20810540 PMC2993457

[B24] Rossner R. Kaeberlein M. Leiser S. F. (2017). Flavin-containing monooxygenases in aging and disease: emerging roles for ancient enzymes. J. Biol. Chem. 292 (27), 11138–11146. 10.1074/jbc.R117.779678 28515321 PMC5500783

[B25] Rui L. (2014). Energy metabolism in the liver. Compr. Physiol. 4 (1), 177–197. 10.1002/cphy.c130024 24692138 PMC4050641

[B26] Schreurs M. Kuipers F. Van Der Leij F. R. (2010). Regulatory enzymes of mitochondrial beta-oxidation as targets for treatment of the metabolic syndrome. Obes. Rev. 11 (5), 380–388. 10.1111/j.1467-789X.2009.00642.x 19694967

[B27] Scott F. Gonzalez Malagon S. G. O'brien B. A. Fennema D. Veeravalli S. Coveney C. R. (2017). Identification of flavin-containing monooxygenase 5 (FMO5) as a regulator of glucose homeostasis and a potential sensor of gut bacteria. Drug Metab. Dispos. 45 (9), 982–989. 10.1124/dmd.117.076612 28646079 PMC5539585

[B28] Stefan N. Haring H. U. Cusi K. (2019). Non-alcoholic fatty liver disease: causes, diagnosis, cardiometabolic consequences, and treatment strategies. Lancet Diabetes Endocrinol. 7 (4), 313–324. 10.1016/S2213-8587(18)30154-2 30174213

[B29] Tan B. L. Norhaizan M. E. (2019). Effect of high-fat diets on oxidative stress, cellular inflammatory response and cognitive function. Nutrients 11 (11), 2579. 10.3390/nu11112579 31731503 PMC6893649

[B30] Tetri L. H. Basaranoglu M. Brunt E. M. Yerian L. M. Neuschwander-Tetri B. A. (2008). Severe NAFLD with hepatic necroinflammatory changes in mice fed trans fats and a high-fructose corn syrup equivalent. Am. J. Physiol. Gastrointest. Liver Physiol. 295 (5), G987–G995. 10.1152/ajpgi.90272.2008 18772365 PMC4059366

[B31] Tong Y. Gao H. Qi Q. Liu X. Li J. Gao J. (2021). High fat diet, gut microbiome and gastrointestinal cancer. Theranostics 11 (12), 5889–5910. 10.7150/thno.56157 33897888 PMC8058730

[B32] Van Herck M. A. Vonghia L. Francque S. M. (2017). Animal models of nonalcoholic fatty liver disease-A starter's guide. Nutrients 9 (10), 1072. 10.3390/nu9101072 28953222 PMC5691689

[B33] Varadi M. Anyango S. Deshpande M. Nair S. Natassia C. Yordanova G. (2022). AlphaFold Protein Structure Database: massively expanding the structural coverage of protein-sequence space with high-accuracy models. Nucleic Acids Res. 50 (D1), D439–D444. 10.1093/nar/gkab1061 34791371 PMC8728224

[B34] Veeravalli S. Varshavi D. Scott F. H. Pullen F. S. Veselkov K. Phillips I. R. (2022). Treatment of wild-type mice with 2,3-butanediol, a urinary biomarker of Fmo5 (-/-) mice, decreases plasma cholesterol and epididymal fat deposition. Front. Physiol. 13, 859681. 10.3389/fphys.2022.859681 36003643 PMC9393927

[B35] Wang S. Yang F. J. Shang L. C. Zhang Y. H. Zhou Y. Shi X. L. (2019). Puerarin protects against high-fat high-sucrose diet-induced non-alcoholic fatty liver disease by modulating PARP-1/PI3K/AKT signaling pathway and facilitating mitochondrial homeostasis. Phytother. Res. 33 (9), 2347–2359. 10.1002/ptr.6417 31273855

[B36] Witayavanitkul N. Werawatganon D. Chayanupatkul M. Klaikeaw N. Sanguanrungsirikul S. Siriviriyakul P. (2020). Genistein and exercise modulated lipid peroxidation and improved steatohepatitis in ovariectomized rats. BMC Complement. Med. Ther. 20 (1), 162. 10.1186/s12906-020-02962-z 32482167 PMC7262771

[B37] Wu Q. Wang Q. Fu J. Ren R. (2019). Polysaccharides derived from natural sources regulate triglyceride and cholesterol metabolism: a review of the mechanisms. Food Funct. 10 (5), 2330–2339. 10.1039/c8fo02375a 31049523

[B38] Xuan T. Liu Y. Liu R. Liu S. Han J. Bai X. (2023). Advances in extraction, purification, and analysis techniques of the main components of kudzu root: a comprehensive review. Molecules 28 (18), 6577. 10.3390/molecules28186577 37764353 PMC10535729

[B39] Yang W. Ling X. He S. Cui H. Yang Z. An H. (2023). PPARα/ACOX1 as a novel target for hepatic lipid metabolism disorders induced by per- and polyfluoroalkyl substances: an integrated approach. Environ. Int. 178, 108138. 10.1016/j.envint.2023.108138 37572494

[B40] Yang W. Liu R. Xia C. Chen Y. Dong Z. Huang B. (2020). Effects of different fatty acids on BRL3A rat liver cell damage. J. Cell Physiol. 235 (9), 6246–6256. 10.1002/jcp.29553 32012270

[B41] Yu M. Alimujiang M. Hu L. Liu F. Bao Y. Yin J. (2021). Berberine alleviates lipid metabolism disorders via inhibition of mitochondrial complex I in gut and liver. Int. J. Biol. Sci. 17 (7), 1693–1707. 10.7150/ijbs.54604 33994854 PMC8120465

[B42] Zhou J. Zhang N. Aldhahrani A. Soliman M. M. Zhang L. Zhou F. (2022). Puerarin ameliorates nonalcoholic fatty liver in rats by regulating hepatic lipid accumulation, oxidative stress, and inflammation. Front. Immunol. 13, 956688. 10.3389/fimmu.2022.956688 35958617 PMC9359096

[B43] Zhou Y. X. Zhang H. Peng C. (2014). Puerarin: a review of pharmacological effects. Phytother. Res. 28 (7), 961–975. 10.1002/ptr.5083 24339367

[B44] Zou Y. Chen Z. Sun C. Yang D. Zhou Z. Peng X. (2021). Exercise intervention mitigates pathological liver changes in NAFLD zebrafish by activating SIRT1/AMPK/NRF2 signaling. Int. J. Mol. Sci. 22 (20), 10940. 10.3390/ijms222010940 34681600 PMC8536011

