# Supplementary material for: Puerarin ameliorates non-alcoholic fatty liver disease by inhibiting lipid metabolism through FMO5
Source: Front Pharmacol. 2024 Jul 11;15:1423634. doi: 10.3389/fphar.2024.1423634 (PMC11269101; doi:10.3389/fphar.2024.1423634)
Supplement: Supplementary file 1 [file DataSheet1.DOCX]

**Supplementary Materials**

**Supplementary Tables**

**Supplementary Table 1. List of primer sequences used in this study**

| Gene | Forward primer | Reverse primer |
| --- | --- | --- |
| *Ho-1* | TGCTAGCCTGGTGCAAGATA | GCCAACAGGAAGCTGAGAGT |
| *Nqo1* | GTCCATTCCAGCTGACAACCA | TTGCCCTGAGGCTCCTAATC |
| *Gclc1* | ATCTGCAAAGGCGGCAAC | ACTCCTCTGCAGCTGGCTC |
| *Cd36* | ATGGGCTGTGATCGGAACTG | GTCTTCCCAATAAGCATGTCTCC |
| *Fabp1* | ATGAACTTCTCCGGCAAGTACC | CTGACACCCCCTTGATGTCC |
| *Fabp4* | AAGGTGAAGAGCATCATAACCCT | TCACGCCTTTCATAACACATTCC |
| *Acc1* | AGGTACAGTAAGAGCCATAGGAC | CTTGGTTGTCAAAATGCCATCAG |
| *Scd1* | TTCTTGCGATACACTCTGGTGC | CGGGATTGAATGTTCTTGTCGT |
| *Fasn* | TAT CCT GCT GTC CAA CCT CAG CAA | TCA CGA GGT CAT GCT TTA GCA CCT |
| *Cpt1α* | GGACTCCGCTCGCTCATT | GAGATCGATGCCATCAGGGG |
| *Pgc1α* | TATGGAGTGACATAGAGTGTGCT | GTCGCTACACCACTTCAATCC |
| *Fmo5* | CCAGTTACGTGAATGATTCG | AGCGCGTGTGAATGCAGGCC |
| *Fmo3* | GGAAGAGTTGGT GAAGACCG | CCCACATGCTTTGAGAGGAG |
| *Ppar-α* | TGCCTTCCCTGTGAACTGAC | TGGGGAGAGAGGACAGATGG |

**Supplementary Figures**


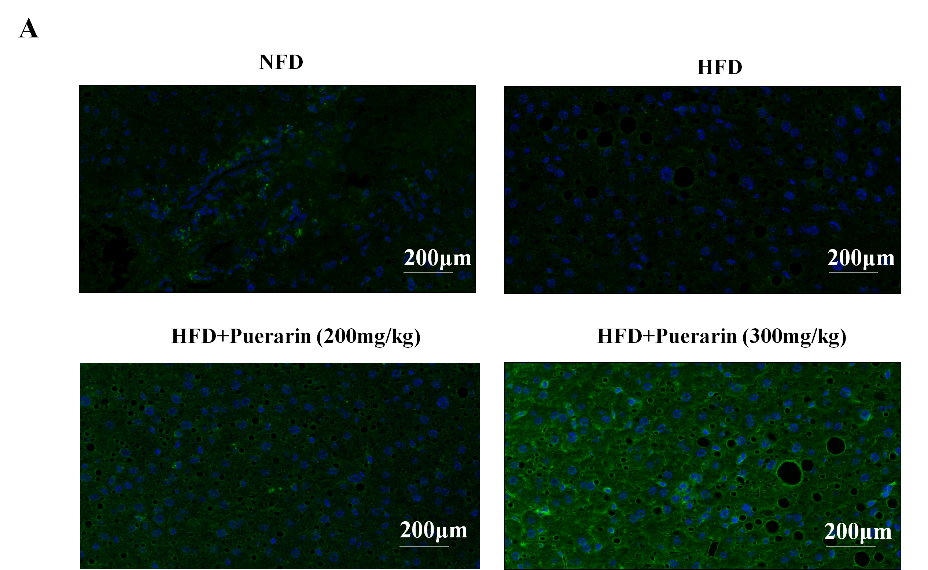


Supplementary Figure 1. The immunostaining of NRF2. (A) Representative immunostaining of NRF2. NRF2 (green, right panel) and DAPI (blue). NFD, normal fat diet; HFD, high-fat diet; HFD200, high-fat diet with puerarin (200 mg/kg/day) intervention; HFD300, a high-fat diet with puerarin (300 mg/kg/day) intervention.


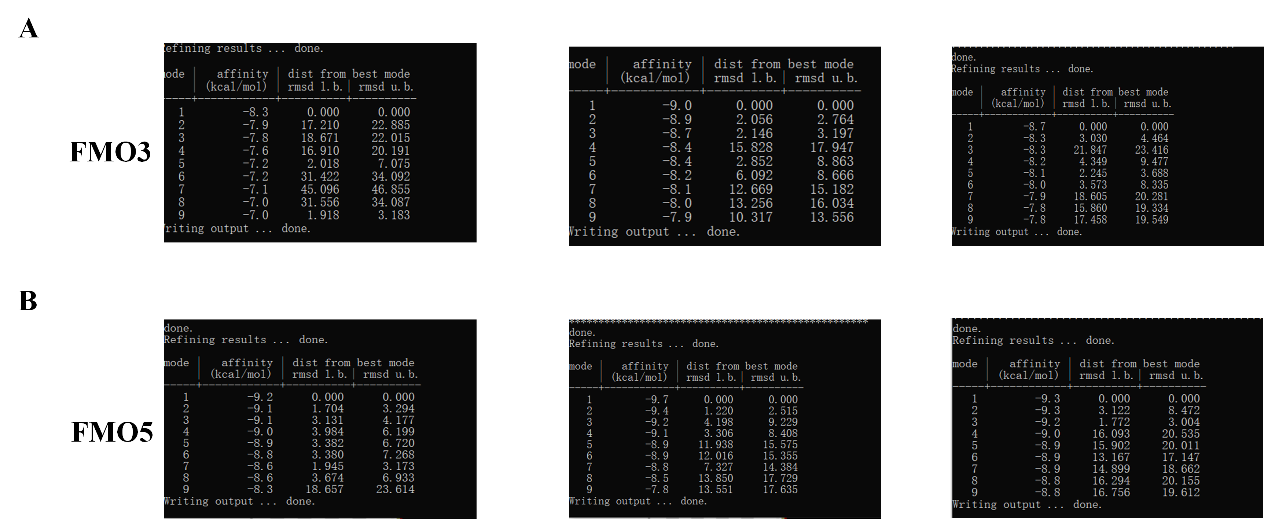


Supplementary Figure 2. The data of docking analysis. (A) the binding energy of docked FMO3 and puerarin. (B) the binding energy of docked FMO5 and puerarin.


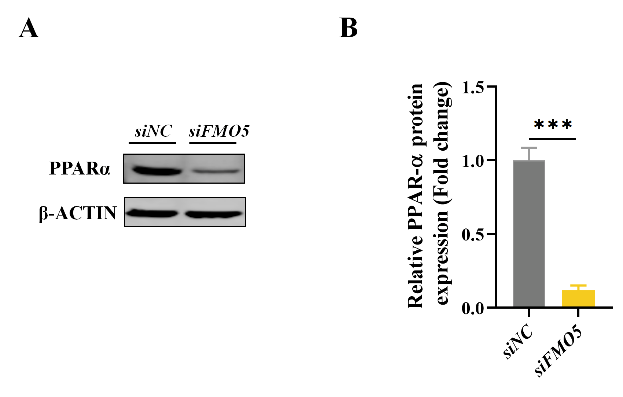


Supplementary Figure 3. Knocking out FMO5 inhibits the expression of PPAR-α. (A-B) PPAR-α protein expression in AML-12 was detected by western blotting and quantification with Image J. * means P < 0.05, ** means P < 0.01, ***means P < 0.001.
